# Prevalence and Associated Risk Factors of Intestinal Parasites among Children under Five Years of Age Attended at Bachuma Primary Hospital, West Omo Zone, Southwest Ethiopia: A Cross-Sectional Study

**DOI:** 10.1155/2023/2268554

**Published:** 2023-05-05

**Authors:** Tadesse Duguma, Teshale Worku, Samuel Sahile, Daniel Asmelash

**Affiliations:** Department of Medical Laboratory Science, College of Health Science and Medicine, Mizan-Tepi University, P.O. Box 260, Mizan-Aman, Ethiopia

## Abstract

**Background:**

In regions of the world with low resources, such as Ethiopia, intestinal parasite diseases are still highly prevalent, especially in children. Poor personal and environmental hygiene, as well as unsafe and low-quality drinking water, are the main causes of this. This investigation aimed to determine the frequency of intestinal parasites and risk factors among children under 5 years age at Bachuma Primary Hospital in 2022.

**Materials and Methods:**

: A cross-sectional study was carried out from October 2022 to December 2022 at Bachuma Primary Hospital, West Omo Zone, Southwest Ethiopia. Stool sample was collected from randomly selected children who were ordered to have their stool examined at the hospital laboratory and wet mount was prepared using normal saline to detect the different stage of intestinal parasites microscopically. Moreover, data related to the sociodemographic and associated risk factors was collected using a structured questionnaire. Descriptive statistics were computed to describe the characteristics of the study participants and determine the prevalence of intestinal parasites. Data were entered into Epi-data manager and analysed using statistical packages for social sciences (SPSS) version 25.0, respectively. Bivariate and multivariate logistic regression analyses were performed, with variables with a *p* value of <0.05 considered statistically significant.

**Result:**

: Infection with at least one intestinal parasite among children was 29.4% (95% CI: 24.5–34.7). *Ascaris lumbricoide* and *Giardia lamblia* were responsible for 8% (26/323) and 4% (13/323) of the prevalence of helminth and protozoans, respectively. A multivariate logistic regression analysis revealed that children whose residence was rural had an adjusted odds ratio (AOR) of 5.048 (*p*=0.001), those who did not wash their hands before meals had an AOR of 7.749 (*p*=0.001), a child with not trimmed fingernails had an AOR of 2.752 (*p*=0.010), a child who frequently experienced stomach pain and whose source of water was pond had an AOR of 2.415 (*p*=0.028) and 3.796 (*p*=0.040), respectively.

**Conclusion:**

In this study, the prevalence of intestinal parasites recorded was low. Rural residency, absence of child hand washing practice before meal, and not trimming fingernail were among factors significantly associated with intestinal parasite infection.

## 1. Introduction

### 1.1. Background

Infections with helminths and protozoa of intestinal parasites are very common worldwide, particularly in developing nations. According to estimates by the World Health Organization (WHO), 3.5 billion people are impacted and 200,000 deaths are reported annually [1]. Intestinal parasites are frequently transmitted by contaminated food or drinking water, but may also be spread from person to person through fecal-oral contact. The prevalence of these parasitic infections varies in different parts of the world [1].

However, in developing nations, the issue is exacerbated by geographic, economic, and unpredictable factors such as natural disasters. The socioeconomic and health of populations with limited resources are significantly affected by intestinal parasites, which are among the most widespread human parasites [2]. Due to many circumstances, the microgeographical distribution of these illnesses may greatly range from one location to another [3].

Today, parasitic infection is a more prevalent and widespread problem due to poor sanitary and affects all people according to conditions, shoe-wearing habits, lack of proper latrines, and other socioeconomic and geographic factors in most regions of the world. Studies conducted in different countries showed that there is a gap in the evaluation of the prevalence of intestinal parasites for children at the primary hospital level. Although intestinal parasite infection is a common public health problem all over the world, it is a more concerning issue in Ethiopia. Wet mount stool examination technique was used to identify parasite species and their prevalence in children under five years at a local primary hospital since wet mount stool examination is the method of choice across health facilities in the country because of its simplicity and affordability.

This study was conducted in West Omo zone, Southwest Ethiopia which is one of the remote/far to reach areas of the country with low coverage of infrastructure and with scanty health facilities where information related to the prevalence of intestinal parasites among vulnerable groups such as children under five year was not sufficiently addressed. So, this study could bring important information on the prevalence of intestinal parasites in these risky population and serve as baseline data for an effective intervention in the study area.

## 2. Materials and Methods

### 2.1. Study Design

A health facility-based cross-sectional study was employed among children under five years who came to Bachuma primary hospital seeking medical services.

### 2.2. Study Area and Period

The study was conducted at Bachuma Primary Hospital from October to December 2022. The study site is found in Bachuma town, which has one public and four private health facilities. The town is in the West Omo Zone, southwest part of Ethiopia, 565 km away from Addis Ababa, the capital city of Ethiopia. Bachuma has high land climate conditions, heavy rainfall, warm temperatures, and a long-wet period. Based on the 2010 census conducted by the CSA, the total population of the town is 3894, of whom 881 are men and 2013 are women. But the hospital health service deliveries covered a larger population and different catchment areas (an estimated population of more than 300,000 from hospital information).

### 2.3. Source Population

Children under five years of age attended at Bachuma Primary Hospital from October to December 2022.

### 2.4. Study Population

Children under five years of age who seek laboratory investigations for intestinal parasites during the study period.

### 2.5. Inclusion and Exclusion Criteria

Children under five years of age who experienced diarrhea during their visit of the hospital laboratory and those without diarrhea but with other related symptoms sent to the laboratory with prescriptions of stool examination during the study period were included whereas, those who had taken antiparasitic drugs in one month period before examination and whose parents/guardians refuse to give sociodemographic and other related data were excluded.

### 2.6. Sample Size and Sampling Technique

#### 2.6.1. Sample Size

The sample size was calculated by using the following formula:

(1) $$\begin{array}{c} n=\frac{\left(\begin{array}{c} Z\alpha /2 \end{array}\right)\;p\left(1-p\right)}{d^{2}}\text{,} \end{array}$$

where *n* is the minimum sample size required, *z* is the critical value for a given confidence interval, and *p* is the expected intestinal prevalence at Bachuma Primary Hospital.

(2) $$\begin{array}{c} n=\frac{\left(\begin{array}{c} Z\alpha /2 \end{array}\right)\;p\left(1-p\right)}{d^{2}}\\ \frac{1.96^{2}\times 0.266\times \left(1-0.266\right)}{0.05^{2}}n\approx 300\text{,} \end{array}$$

where *n* represents minimum sample size, *p* represents prevalence rate (26.6%) [4] taken from a study conducted on intestinal parasites among children under five years at public health facilities in Hawassa, (*Zα*/2) represents standards normal variable (1.96), and *d* represents margin of sampling error (5%).

Adding a 10% nonresponse rate, the final sample size was (*n*) 300 + 30.0 = 330.

#### 2.6.2. Sampling Technique

A systematic random sampling technique was employed to select the children to participate in the study from those sent to the laboratory department of the hospital for stool examination. The sampling frame was obtained from the registered information by the different health facilities in the catchment of the area where this study was conducted and accordingly there were about 1286 children under the age of five years who got services such as immunization/vaccination at different health facilities in the catchment. The *K*^th^ interval calculated from the population and the sample size was (3.89 ≈ 4). Hence, the study participants (only children under five years) who met the inclusion criteria were picked every fourth after randomly selecting the first child by lottery system until the desired sample size was reached. Finally, 323 children who fulfil the inclusion criteria were included in the study.

## 3. Data Collection and Analysis

### 3.1. Data Collection

One gram (1 gm) of stool sample was collected from each child whose parents or guardians signed an informed written consent form and expressed a desire to participate in the study. A pretested structured questionnaire was used to collect data related to sociodemographic characteristics and other independent variables. The data generated from the laboratory results and questionnaires were summarized for the final analysis.

### 3.2. Stool Wet Mount Technique

Wet mount stool sample examination is the most used method of intestinal parasite detection in almost all the health facilities in Ethiopia. This method is simple and easy to perform with little financial investment compared to other techniques such as concentration technique and molecular level tests but with low sensitivity. The wet mount slide was prepared taking 2 milligrams of either diarrheic or nondiarrheic stool sample from the children and the slides were examined microscopically under both 10x and 40x objectives of light microscope by two laboratory technicians to identify the different stages of protozoans and helminths. To prepare the wet mount, a microscope slide, cover slip, and a drop or two drop of normal saline were used. Thickness of the wet mount was made to the standard to enable detection of all the possible stages of intestinal parasites.

### 3.3. Data Analysis

Epi data was used to enter data and analysed using SPSS version 25. Descriptive statistics were used to show the prevalence of intestinal parasites. Tables and figures were used to present the data. Binary logistic analysis was performed first, and variables with a *p* value <0.25 were considered candidates for multivariable logistic regression for the final analysis. An adjusted odds ratio (AOR) with a 95% confidence interval (CI) was reported. In the multivariable logistic regression analysis, variables with a *p* value of less than 0.05 were significantly associated with intestinal parasites infection.

### 3.4. Data Quality Control

The standard operating procedure (SOP) was strictly followed for the stool sample examination process. To avoid a technical error that encountered the quality of normal saline was checked during sample collection and microscopic examination, that is, internal quality control was conducted. The data collection format of each data was checked daily for completeness of missed or other relevant data during data collection, as well as by the principal investigators. Special emphasis was given to diarrhea stool samples. The consistency and completeness were checked by the investigators and the questionnaire was pretested at the Mizan-Tepi Teaching Hospital.

To ensure the quality of the investigation, two laboratory technicians examine the slides independently and their readings were compared. Discordant results were immediately resolved with a discussion among them and in consultation with an experienced laboratory expert.

## 4. Result

### 4.1. Sociodemographic Characteristics

A total of 323 children with an age range of 6 months to 59 months and each child's parents or caregivers participated in this study. More than half of the children were female, representing a higher proportion of intestinal parasite infections than their male counterparts. Intestinal parasite infection was relatively higher in the age category of 12–36 months compared to those under 1 year and over 3 years. Out of 323 parents/guardians who participated in this study, 43.7% of them earn 500–1000 Ethiopian Birrs monthly. Of the total study participants, 28.2% were illiterate and among daily laborers, a relatively high number of parents/caregivers participated in the study (Table 1).

### 4.2. Intestinal Parasite Prevalence

**Figure 1:**
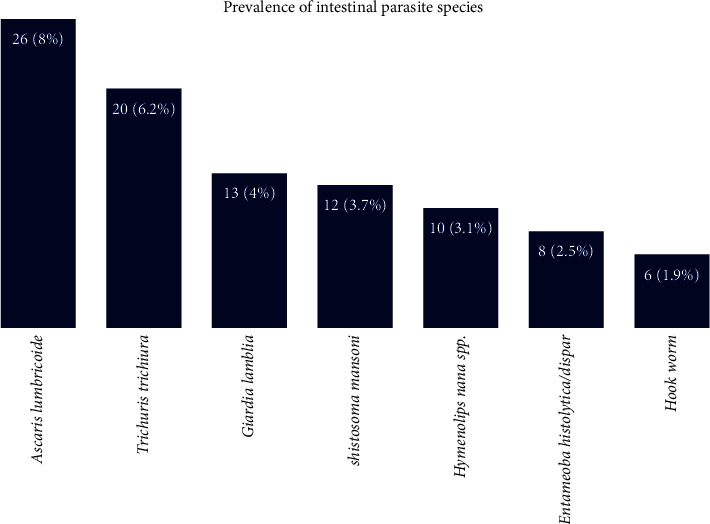
Intestinal parasites prevalence by species at Bachuma primary hospital, Southwest Ethiopia.

The prevalence of intestinal parasites in children under five years of age in the study was recorded as 29.4% (95/323). A total of 9.9% (32/323) coinfections were recorded in this study. The coinfection showed *Ascaris lumbricoide* with *Trichuris trichiura* (3.4%), *T. trichiura* with *Schistosoma mansoni* (2.8%), Hookworm and *A. lumbricoide* (1.5%), *Schistosoma mansoni* and *E. histolytica/dispar* (1.2%), and *E. histolytica/dispar* and Hookworm (0.9%). Seven species of intestinal parasites were identified, and of them, *Ascaris lumbricoide* was the most common intestinal parasite detected, followed by *Trichuris trichiura*, *Giardia lamblia*, *Schistosoma mansoni*, *H. nana* spp., *E. histolytica/dispar*, and Hookworm, respectively (Figure 1).

In this study, two-thirds of the houses were found to be uncemented, and more than two-thirds of caregivers/mothers did not wash their hands with soap and clean water before feeding their child, but more than half of them did so after using the toilet. More than one-third of the children in this study use water from a stream, and more than half of them have trimmed fingernails. More than three-fourths of the children ate vegetables and fruits that were not washed (Table 2).

### 4.3. Intestinal Parasite Infections-Related Factors

All variables with a *p* value of <0.25 were taken to the multivariate logistic regression model accordingly. Some of the associated factors showed a significant association with the infection of intestinal parasites in a child: children whose residence was in rural AOR (5.048; 95% CI: 2.166–11.764) were more likely to be infected with intestinal parasites than those from urban, those who did not wash their hands before meal AOR (7.749; 95% CI: 2.684–22.373) were 7.749 times more likely to contract intestinal parasite infection compared to their counterparts. Moreover, children whose fingernails were not trimmed and who experienced stomach pain frequently were more than twofold more susceptible to intestinal parasitic infection with AOR (2.752; 95% CI: 1.280–5.917) and (2.415; 95% CI: 1.100–5.302), respectively. The source of drinking water appeared to play a significant role in increasing the risk of intestinal parasite infection, with those who used water from a pond have AOR of (3.796; 95% CI: 1.060–13.591) (Table 3).

### 4.4. Prevalence of Intestinal Parasites in Relation to Certain Clinical Symptoms

**Figure 2:**
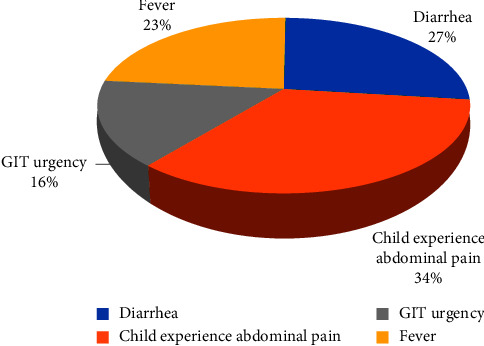
Prevalence of intestinal parasites in relation to various clinical symptoms in children under five years.

More than one-third of the study participants/children experienced abdominal pain before stool sample collection, and more than one-fourth of the children also had diarrhea during the study period. Those with gastrointestinal tract urgency accounted for 16% of the participants (Figure 2).

## 5. Discussion

Successful preventive and therapeutic interventions towards intestinal parasitic infections emanates from understanding of the distribution and extent of intestinal parasitic infection in a given community. This study assessed the prevalence as well as the associated factors of intestinal parasite infection. The overall prevalence of intestinal parasitic infections was 29.4%, of which children in the age category of 12–36 months takes relatively the highest share. The findings of this study were consistent with those of other studies conducted in Ethiopia; specifically, what was discovered in Bair Dar, northwest Ethiopia (24.4%) [5], Wonji Shoa Sugar Estate, Ethiopia (24.3%) [6] and Rural Dembiya Ethiopia (25.4%) [7] showed this consistency. The similarity in prevalence between the current study and those mentioned above could be because the study using similar inclusion criteria for the recruitment of study participants. But compared to several studies carried out in various parts of the world, the current study revealed a low prevalence of intestinal parasite infection at Yirgalem Hospital, Ethiopia (36.52%) [8], North Sumatera, Indonesia (34.4%) [9], Bale Zone, Ethiopia (38.5%) [10], Dera District, Boricha district, South Ethiopia (48.7%) [11], Tigray region, northern Ethiopia (58%) [12], Northwest Ethiopia (62.3%) [13], a tertiary care hospital in Karachi, Pakistan (68.8%) [14], and Wondo Genet, southern Ethiopia (85.1%) [15]. The low prevalence recorded in this study could be due to the method used for intestinal parasite detection. Only wet mount preparation was used as the main diagnostic technique, which may contribute to the low prevalence, while in some of the above studies, better detection techniques such as concentration technique was used which could increase the prevalence. The finding of this study is higher than what is found at Dessie Referral Hospital (15.5%) [16], Debre Birhan referral hospital, North Shoa, Ethiopia (17.4%) [17], Debre Tabor Comprehensive Specialized Hospital, Northwest Ethiopia (17.44%) [18], Mekane Eyesus Primary Hospital, Northcentral Ethiopia (18.0%) [19], Woreta Health Center, Northwest Ethiopia (18.7%) [20], Bahir Dar and Han Health Centers, Northern Ethiopia (19%) [21], and South Ethiopian Hospital (21.2%) [22]. The reason for these variations may be attributed more to the difference in geographical locations of the study areas, that is, the current study was conducted in relatively less urbanized parts of the country with low coverage infrastructures such as health facilities. This study revealed that children from rural areas were more likely to have intestinal parasite infections than children from urban areas, and those who did not wash their hands before meals were 7.749 times more likely to contract an intestinal parasite infection compared to their counterparts. This might be due to the low coverage of deworming services in rural areas compared to urban dwellers. Moreover, children whose fingernails were not trimmed and who experienced stomach pain frequently were more than twice as susceptible to intestinal parasitic infection. Those children whose fingernails were not shortened or trimmed were 2.752 times prone to intestinal parasite infection contributing 18% of the prevalence, which is supported by similar studies conducted in Ethiopia [23]. This could be attributed to the lack removal of accumulated dirt containing parasite eggs in fingernails which could serve as source infection [24]. The source of drinking water also appeared to play a great role in increasing the chance of getting an intestinal parasite infection, hence children who used water from pond accounted for a relatively high number of the positive cases. This might be due to contamination of water and food with human waste during open field defecation. In addition, lack of awareness about the importance of hand washing practices after defecation can lead to easy contamination of people through food. According to a study from Côte d'Ivoire; poor sanitation and hygiene practices are linked to intestinal protozoa and helminth infections that are transferred through the soil [25]. The distribution and prevalence of various species of intestinal parasites differ from region to region because of several environmental, social, and geographical and other factors. In this study, the wet mount microscopy method was used to determine the prevalence of intestinal parasites although molecular assays and concentration techniques could best estimate the burden of intestinal parasites.

### 5.1. Limitation of the Study

Molecular tests and other concentration techniques could best estimate the prevalence of intestinal parasites compared to the conventional microscopy method which has low sensitivity. Furthermore, differentiation between the morphologically identical species of *Entamoeba* was not within the scope of this study, as only wet mount microscopy was used.

## 6. Conclusion and Recommendation

A low prevalence of intestinal parasite infection was recorded among the children under five years of age who participated in this study. *Ascaris lumbricoide*, *Trichuris trichiura*, and *Giardia lamblia* were the most common parasites found in the study. Rural residency, not washing hands before meal, not trimming fingernails, and using unclean water (from streams and ponds) were some of the factors significantly associated with intestinal parasitic infection. Diagnostic techniques with better detection ability such as formol-ether concentration technique and molecular based tests could offer better result than what is found by wet mount in this study.

## Figures and Tables

**Table 1:** Characteristics of children under five years and caretakers/guardians in terms of sociodemographic variables in Bachuma primary hospital, Southwest Ethiopia, 2022 (*n* = 323).

Variable	Category	Frequency	Percent
Age (months)	6–11	79	24.5
	12–36	155	48
	37-59	89	27

Sex	Male	144	44.6
	Female	179	55.4

Residence	Urban	175	54.2
	Rural	148	45.8

Educational status of the guardians (grade)	Illiterate	91	28.2
	1–4	39	12.1
	5–8	67	20.7
	9–12	77	23.8
	Diploma and above	49	15.2

Occupational status of the guardians	Farmer	71	22
	Housewives	81	25.1
	Daily laborer	88	27.2
	Merchant	44	13.6
	Employed	33	10.2
	Other	48	14.9

Monthly income of the parents/caregivers (Ethiopian birr)	<500	88	27.2
	500–1000	141	43.7
	1001–2000	56	17.3
	>2000	38	11.8

**Table 2:** Intestinal parasite infection risk factors in children under five years age attended at Bachuma primary hospital, Southwest Ethiopia (*n* = 323), 2022.

Variable	Category	Frequency	Percent
Floor of the house	Cemented	108	33.4
	Not cemented	215	66.6

Washing hand with clean water and soap before meal	Yes	105	32.5
	No	218	67.5

Washing hand after toilet	Yes	181	56
	No	142	44

Children infected with IP before	Yes	147	45.5
	No	176	54.5

If yes how long	<2 weeks	7	2.2
	>2 weeks	6	1.8
	<1 month	52	16.1
	>1 month	74	22.9
	Not infected before	184	57

The child's fingernails trimmed	Yes	172	53.3
	No	151	46.7

Vegetable/fruit washed before eating	Yes	74	22.9
	No	249	77.1

Did your child's get treated for GIT infection before?	Yes	168	52
	No	155	48

Did your child has taken any medication in the last 15 days	Yes	130	40.2
	No	193	59.8

If yes what type of drug	Antihelminth	53	16.4
	Antiprotozoal	45	13.9
	Antibiotics	32	9.9
	Medication not taken	193	59.8

Source water	Stream	113	35
	Pond	70	21.7
	Tap water	81	25.1
	Others	59	18.2

Total		323	100

**Table 3:** Analysis of related risk factors for intestinal parasite infection in children under five years of age using bivariate and multivariate logistic regression at Bachuma primary hospital, Ethiopia (*n* = 323), 2022.

Variable	Category	Frequency *n*(%)	Positive for IP *n*(%)	Odds ratio (95% CI)			*p* value
				COR	*p* = value	AOR	
Age (months)	6–11	79 (24.4)	16 (5.0)	Ref		Ref	
	12–36	155 (48.0)	45 (13.9)	0.621 (0.324, 1.188)	0.150	1.162 (0.393, 3.433)	0.786
	37-59	89 (27.6)	34 (10.5)	0.411 (0.205, 0.824)	0.012	2.041 (0.894, 4.660)	0.090

Sex	Male	144 (44.6)	44 (13.6)	1.072 (0.765, 1.504)	0.686		
	Female	179 (55.4)	51 (15.8)	Ref		Ref	

Residence	Urban	175 (54.2)	28 (8.7)	Ref		Ref	
	Rural	148 (45.8)	67 (20.7)	0.353 (0.241, 0.518)	0.001	5.048 (2.166, 11.764)^*∗*^	**0.001**

Floor of the house	Cemented	108 (33.4)	26 (8.0)	Ref		Ref	
	Uncemented	215 (66.6)	69 (21.4)	0.750 (0.509, 1.105)	0.136	1.445 (0.668, 3.127)	0.350

Child wash hand before meal	Yes	105 (32.5)	8 (2.5)	Ref		Ref	
	No	218 (67.5)	87 (26.9)	0.191 (0.096, 0.379)	0.001	7.749 (2.684, 22.373)^*∗*^	**0.001**

Child wash hand after toilet use	Yes	172 (53.3)	40 (12.4)	Ref		Ref	
	No	151 (46.7)	55 (17.0)	0.638 (0.453, 0.900)	0.010	1.926 (0.913, 4.060)	0.085

Child infected with IP before	Yes	147 (45.5)	46 (14.2)	1.124 (0.802, 1.575)	0.498		
	No	176 (54.5)	49 (15.2)	Ref		Ref	

Child's fingernails trimmed	Yes	181 (56.0)	37 (11.5)	Ref		Ref	
	No	142 (44.0)	58 (18.0)	0.500 (0.353, 0.709)	0.001	2.752 (1.280, 5.917)^*∗*^	**0.010**

Vegetable/Fruit washed before eating	Yes	74 (22.9)	19 (5.9)	Ref		Ref	
	No	249 (77.1)	76 (23.5)	0.841 (0.547, 1.294)	0.422		

Child experienced stomach pain frequently	Yes	198 (61.3)	71 (22.1)	1.868 (1.246, 2.800)	0.001	2.415 (1.100, 5.302)^*∗*^	**0.028**
	No	125 (38.7)	24 (7.4)	Ref		Ref	

Child have diarrhea	Yes	239 (74.0)	68 (21.1)	Ref		Ref	
	No	84 (26.0)	27 (8.4)	0.885 (0.611, 1.282)	0.523		

Child's GIT infection treated before	Yes	168 (52.0)	50 (15.6)	1.025 (0.731, 1.438)	0.886		
	No	155 (48.0)	45 (13.9)	Ref		Ref	

Child has taken any medication in the last 15 days	Yes	130 (40.2)	38 (11.8)	Ref		Ref	
	No	193 (59.8)	57 (17.6)	0.990 (0.701, 1.398)	0.953		

Source water	Stream	113 (35.0)	38 (11.8)	0.310 (0.133, 0.718)	0.006	2.786 (0.823, 9.432)	0.100
	Pond	70 (21.7)	26 (8.0)	0.265 (0.109, 0.646)	0.003	3.796 (1.060, 13.591)^*∗*^	**0.040**
	Tap water	81 (25.1)	23 (7.1)	Ref		Ref	
	Others	59 (18.3)	8 (2.5)	2.396 (0.163, 0.961)	0.041	2.460 (0.653, 9.270)	0.184

Total		323 (100)	95 (29.4)				

Note : *p* < 0.05 was taken as significance, Ref stands for reference category.

## Data Availability

The data related to this study are available from the corresponding author upon reasonable request.

## Abbreviations

IP: Intestinal parasite
IPIS: Intestinal parasitic infections
NGO: Non-Governmental Organization
WHO: World Health Organization
BPH: Bachuma primary hospital
SOP: Standard operating procedure
LPF: Low-power field
HPF: High-power field
KAP: Knowledge, attitude, and practice.

## Supplementary Materials

Supplementary Materials A structured questionnaire was prepared in English and translated to the local language spoken by the study participants prior to data collection. It aims to assess the knowledge status of parents/guardians of children under five years who attended at Bachuma primary hospital, from October to December, 2022. The main components of the questionnaire were variables related to the sociodemographic characteristics of the children such as age, sex, and resident; sociodemographic characteristics of the parents/guardians (age, sex, resident, educational status, occupational status, and family monthly income); and questions related to associated risk factors of intestinal parasitic infection and clinical manifestation of children under the age of five.
