# Supplementary material for: Prevalence and Associated Risk Factors of Intestinal Parasites among Children under Five Years of Age Attended at Bachuma Primary Hospital, West Omo Zone, Southwest Ethiopia: A Cross-Sectional Study
Source: J Trop Med. 2023 May 5;2023:2268554. doi: 10.1155/2023/2268554 (PMC10181902; doi:10.1155/2023/2268554)
Supplement: Supplementary Materials — A structured questionnaire was prepared in English and translated to the local language spoken by the study participants prior to data collection. It aims to assess the knowledge status of parents/guardians of children under five years who attended at Bachuma primary hospital, from October to December, 2022. The main components of the questionnaire were variables related to the sociodemographic characteristics of the children such as age, sex, and resident; sociodemographic characteristics of the parents/guardians (age, sex, resident, educational status, occupational status, and family monthly income); and questions related to associated risk factors of intestinal parasitic infection and clinical manifestation of children under the age of five. [file 2268554.f1.pdf]

ANNEX-I: English Questionnaire

Mizan Tepi University College of Medicine and Health Sciences College Department of Medical Laboratory Sciences.

This questionnaire is prepared to assess the prevalence of intestinal parasite infection among children five years who attended at Bachuma primary hospital, October to December, 2022. So, you are invited to give valuable information about your child and I also guaranteed all information you give for this study will be kept confidential. Finally, I ask your cooperation and patience until I finish my question.

Are you voluntary? A. Yes ☐ B. No ☐

Data collection format Part-I: Participant identification

1. Age ____________
2. Sex A. Male ☐ B. Female ☐
3. Residence A. Urban ☐ B. Rural ☐
4. Educational status of parents or guardians
   A. 1-4 ☐ B. 5-8 ☐
   B. 9-12 ☐ D. Diploma and above ☐
5. Occupational status of parents or guardian
   A. Farmers ☐ D. Daily laborer ☐ G. Others ☐
   B. Housewives ☐ E. Merchant ☐
   C. Student ☐ F. Employed ☐
6. Monthly income of the parents/guardians
   A. < 500 birrs ☐ C. 1001- 2000 birrs ☐
   B. 500-1000 birrs ☐ D. > 2000 birrs ☐

Part II: Associated factors

7. Floor of the house and the compound in which the child play
   A. cemented ☐ B. made of mud/uncemented ☐

8. Did you wash your hand with soap and clean water before meal?

A. Yes ☐ B. No ☐

9. Did you wash your hands after toilet?

A. Yes ☐ B. No ☐

10. Has your child infected with intestinal parasite before?

A. Yes ☐ B. No ☐

11. If yes, how long it was? A. < 2 weeks ☐ B. > 2 weeks ☐

C. < 1month ☐ D. > 1month ☐

12. Child's finger nail trimmed/cut short

A. Yes ☐ B. No ☐

13. Vegetables/Fruits was eaten

A. Washed well ☐ B. Not washed ☐

14. Have your child get treated for GIT infection before?

A. Yes ☐ B. No ☐

15. Did your child have abdominal pain problem?

A. Yes ☐ B. No ☐

16. Did your child was a diabetic Patient?

A. Yes ☐ B. No ☐

17. Did your child take any medication in the last 15 days?

A. Yes ☐ B. No ☐

18. If yes what type of drug? A. Antihelminth ☐ B. Antiprotozoal ☐ C. Antibiotics ☐

19. Source of water for drink for the family

A. Stream ☐ B. Pond ☐ C. Pipe water ☐ D. Others ☐

Part III: Clinical Manifestation of the child (Put√)

20. Diarrhea: A. Yes ☐ B. No ☐

21. Stomach pain Frequently: A. Yes ☐ B. No ☐

22. GIT Urgency: A. Yes ☐ B. No ☐

23. Fever A. Yes ☐ B. No ☐
